# Oral health and obesity indicators

**DOI:** 10.1186/1472-6831-12-50

**Published:** 2012-11-20

**Authors:** Anna-Lena Östberg, Calle Bengtsson, Lauren Lissner, Magnus Hakeberg

**Affiliations:** 1 Dept of Behavioral and Community Dentistry, Institute of Odontology, The Sahlgrenska Academy, University of Gothenburg, Göteborg, Sweden; 2 Research Center, Public Dental Service, Region Västra Götaland, Göteborg, Sweden; 3 Dept of Public Health and Community Medicine/Primary Health Care, The Sahlgrenska Academy, University of Gothenburg, Göteborg, Sweden; 4 Dept of Public Health and Community Medicine/Public Health Epidemiology, The Sahlgrenska Academy, University of Gothenburg, Göteborg, Sweden

**Keywords:** Body mass index, Waist-to-hip ratio, Waist circumference, Number of teeth, Health behaviour

## Abstract

**Background:**

In western Sweden, the aim was to study the associations between oral health variables and total and central adiposity, respectively, and to investigate the influence of socio-economic factors (SES), lifestyle, dental anxiety and co-morbidity.

**Methods:**

The subjects constituted a randomised sample from the 1992 data collection in the Prospective Population Study of Women in Gothenburg, Sweden (n = 999, 38- > =78 yrs). The study comprised a clinical and radiographic examination, together with a self-administered questionnaire. Obesity was defined as body mass index (BMI) > =30 kg/m^2^, waist-hip ratio (WHR) > =0.80, and waist circumference >0.88 m. Associations were estimated using logistic regression including adjustments for possible confounders.

**Results:**

The mean BMI value was 25.96 kg/m^2^, the mean WHR 0.83, and the mean waist circumference 0.83 m. The number of teeth, the number of restored teeth, xerostomia, dental visiting habits and self-perceived health were associated with both total and central adiposity, independent of age and SES. For instance, there were statistically significant associations between a small number of teeth (<20) and obesity: BMI (OR 1.95; 95% CI 1.40-2.73), WHR (1.67; 1.28-2.19) and waist circumference (1.94; 1.47-2.55), respectively. The number of carious lesions and masticatory function showed no associations with obesity. The obesity measure was of significance, particularly with regard to behaviour, such as irregular dental visits, with a greater risk associated with BMI (1.83; 1.23-2.71) and waist circumference (1.96; 1.39-2.75), but not with WHR (1.29; 0.90-1.85).

**Conclusions:**

Associations were found between oral health and obesity. The choice of obesity measure in oral health studies should be carefully considered.

## Background

The prevalence of obesity has increased internationally over the last decades, and was in the 1990s considered by the World Health Organization as a global epidemic [1]. The link between obesity and a series of diseases has been confirmed, particularly cardiovascular diseases and diabetes [2]. Studies also indicate poor oral health in obese people [3]. For instance, more teeth had been lost and periodontal disease was found more frequently in obese individuals [4,5].

Obesity is generally measured by means of body mass index (BMI, weight in kilogram divided by height in metres^2^), an overall measure of general obesity [1]. Waist circumference [6] and waist-hip ratio (WHR) are used as indicators of centralised fat distribution [7,8]. All these variables have been identified to be related to a series of diseases, such as diabetes and cardiovascular disease [8,9]. However, the association with WHR was less consistent and more uncertain [10]. In studies of oral health, different measures of obesity are less well investigated [4].

General health and oral health share similar causal and behaviour mechanisms [11], and the self-perceived oral health of an individual has been related to general health [12]. For instance, dental attendance patterns are correlated with other health habits [13]. In turn, irregular dental care was found to be associated with dental anxiety [5].

A number of common possible confounders should be considered in studies of oral health and obesity. Among those are socio-economic and lifestyle factors which are associated with both body weight [14] and oral health [11].

The aim of the current study was to study the associations between oral health variables and total and central adiposity, respectively. A second aim was to investigate whether these associations were dependent of socio-economic factors, lifestyle, dental anxiety and co-morbidity.

## Methods

The sample comprised subjects from The Prospective Population Study of Women in Gothenburg, initiated in 1968 [15]. A randomised sample from the general population of Gothenburg (1622 women, ages 38, 46, 50, 54, 60 yrs) was invited for a combined medical, psychiatric and dental examination. The participation rate was 90.1%, with 1462 women participating (90.1%). They were re-examined in 1980–81 [16] and in 1992–93 [17].

Data from the 1992–93 investigation were used in the current study. New cohorts, born in 1942 and 1954 and randomly selected from the population of Gothenburg, were included. A few women born in 1922 and 1930, who had moved to Gothenburg after 1968, were also randomly selected and included in the study to ensure representativeness in the different age strata [17]. Of the initial participants, 57.2% participated in the 1992–1993 study (280 deceased, 89 moved away, 255 declined) [17]. The overall participation rate was 70.1% [18]. The analyses in this study are based on medical and dental information gathered from a total of 999 women. Information on socio-economic status, health-related lifestyle habits including dental health habits and dental anxiety was obtained using self-reported questionnaires. The study was approved by the Research Ethics Committee of the University of Gothenburg and informed consent was obtained from all participants.

In the medical part of the study, a physical examination was carried out together with a collection of fasting blood samples. The women were weighed to the closest 0.1 kg wearing only briefs, and their height (no shoes) was measured to the nearest 0.5 cm. The women’s waist circumference was determined using a steel tape measure, midway between the lower rib margin and the iliac crest, and the hip circumference was measured at the widest point between the iliac crest and the buttock. The circumferences were measured with the subject in the standing position and to the nearest 1 mm [19].

The patients were summoned to one special dental unit where the dental examinations were conducted by two specially trained and calibrated dentists. It comprised a clinical inspection of the teeth and the oral mucosa together with a panoramic radiography. The number of teeth was recorded 1–32, as were the number of restored teeth. The number of manifest dental caries lesions was clinically determined or radiographically diagnosed when reaching the dentin. Information on dental visiting habits (twice a year/once a year/each second year/occasionally/acute/never), the last dental visit (<1 year/1-2 years/3-5 years/>5 years ago), xerostomia (no/one week/one month/6 months/>6 months), masticatory function (very poor/rather poor/neither bad or good/rather good/very good) was collected by means of the questionnaire.

### Variables

The dependent variables were three separate measures of obesity. General obesity was represented by body mass index (BMI, weight in kilogram/(height in metres^2^)) and participants were defined as obese when their BMI was ≥30 kg/m^2^[1]. Abdominal obesity was diagnosed when the waist circumference was >88 cm [6]. Furthermore, the waist circumference was divided by the hip circumference to give a ratio, the waist-to-hip ratio (WHR). The subjects were defined as obese when their WHR was ≥0.80 [10].

The independent variables were age (used as a continuous variable), number of teeth (continuous variable and categorised for various analyses 0, 1–9, 10–19 and ≥20, respectively), number of restored teeth (continuous variable), carious lesions (≥1 versus 0), time since latest dental visit (≥1 year versus <1 year), and dental visiting habits (<once a year versus ≥ once a year). Self-perceived oral health was assessed by prolonged xerostomia (≥six months versus < six months) and masticatory function (measured on a scale of 1–5, dichotomised as poor (1-3) or acceptable (4,5).

Possible confounders included socio-economic status, lifestyle variables, dental anxiety and co-morbidity. Socio-economic status was represented by marital status (married, unmarried) together with subjective evaluations of the subjects’ economic and social situation, both measured on a scale of 1–7 and dichotomised as poor (1-4) or acceptable (5-7). Lifestyle was measured using smoking (smoker, non-smoker), alcohol habits (some times a week or daily versus ≤ once a week), physical activity (sedentary, some, regular moderate, regular heavy) and participation in cultural activities (no/yes) as variables. Dental anxiety was measured using Corah’s Dental Anxiety Scale (DAS; possible scores 4–20, dichotomised into ≥13 versus <13) [20].

Co-morbidity was represented by angina pectoris and/or a history of myocardial infarction, hypertension and diabetes. Angina pectoris was defined according to Rose [21]. Myocardial infarction was diagnosed when two or more of the following criteria were fulfilled: i) central chest pain >30 minutes, ii) transient rise of transaminase activities, and iii) typical electrocardiogram changes of recent onset [22]. Hypertension was dichotomised as yes or no, where “yes” was recorded when the subject had a systolic blood pressure ≥160 mm Hg and/or a diastolic blood pressure ≥95 mm Hg and/or was treated pharmacologically for hypertension. Diabetes was likewise dichotomised as yes or no, where “yes” was recorded if the subject was on anti-diabetes therapy (injecting insulin and/or having oral medication) or if two fasting blood samples showed elevated glucose concentrations according to the definition by the World Health Organization (≥7.0 mmol/l).

### Statistical analysis

The statistical analyses were carried out using the SPSS software, version 18.0. The analyses included descriptive statistics giving percentages, means and standard deviations. Pearson’s correlation analysis was used to study the interrelationship between different measures of obesity. Associations between independent and dependent variables were tested using logistic regression analysis and expressed as odds ratios (OR) with 95% confidence intervals (CI). Possible confounders were introduced as covariates in multivariate models. Interaction terms between age and number of teeth and between dental anxiety and dental visiting habits, respectively, were explored in relation to obesity. Results were considered to be statistically significant when *p* < 0.05 or when CI excluded 1.

## Results

The mean age of the subjects was 65.3 years (SD 10.7). Mean values and standard deviations for anthropometric values (BMI, WHR and waist circumference) in the different age cohorts are shown in Table 1. In the total group, the mean BMI was 25.96 (SD 4.25), the mean WHR 0.83 (SD 0.06) and the mean waist circumference 83.0 cm (SD 11.06).

**Table 1:** Age distribution in cohorts related to anthropometric measures

**Age**	**n**	**BMI**	**WHR**	**Waist**
		**Mean (SD)**	**Mean (SD)**	**Mean (SD)**
38	67	23.5 (3.3)	0.80 (0.05)	76.1 ( 9.1)
50	98	25.0 (4.0)	0.81 (0.07)	80.1 (12.3)
62	269	26.5 (4.4)	0.83 (0.06)	84.0 (10.8)
70	276	26.1 (4.1)	0.83 (0.06)	83.4 (10.5)
74	203	26.4 (4.4)	0.83 (0.06)	84.6 (11.2)
≥78	86	25.9 (4.2)	0.83 (0.06)	83.6 (10.8)

Characteristics for independent variables (clinical and self-perceived dental variables) and covariates (co-morbidity, SES and lifestyle) are given in Table 2. The mean number of teeth decreased steadily with age, from 28.1 (SD 2.2) in 38-year-olds to 12.5 (SD 9.1) in those 78 years and older. A total of 126 individuals had no own teeth left. The interaction terms between the number of teeth and age were borderline statistically significant, independent of the teeth cut-off number (not in tables).

**Table 2:** Characteristics of the study population in independent variables and covariates

	**Mean**	**SD**
No of teeth (range 0 – 32)	17.4	9.5
No of restored teeth (range 0–31)*	15.3	6.9
No of carious lesions (range 0–10 )*	0.3	0.9
	**n**	**%**
Dental visit habits (< once a year)	173	17.4
Latest dental visit (> one year)	207	20.8
Xerostomia (longstanding)	307	31.2
Masticatory function (poor)	200	20.2
Co-morbidity		
Hypertension (yes)	453	48.7
Angina pectoris and/or myocardial infarction (yes)	69	7.0
Diabetes (yes)	50	5.0
Socio-economic factors		
Marital status (unmarried)	436	44.0
Social situation (poor)	137	13.8
Economic situation (poor)	253	25,5
Health situation (poor)	436	43.9
Lifestyle		
Smoking (current)	202	20.5
Alcohol (several times a week)	234	23.5
Culture activities (not participating)	318	32.6
Regular physical activity (no)	203	20.4

* edentulous individuals excluded (n = 126).

Missing cases 0.2 – 6.9.

The correlations between BMI and WHR was r = 0.23, between BMI and waist circumference r = 0.59 and finally, between WHR and waist circumference r = 0.41.

The number of teeth showed strong and consistent statistically significant associations with obesity; however the pattern varied with different obesity measures (BMI, WHR and waist circumference, respectively) (Table 3). A larger number of teeth (continuous variable) showed an overall trend towards a lower risk of obesity, irrespective of the obesity measure: the crude OR (95% CI) for BMI was 0.96 (0.95-0.98), for WHR 0.97 (0.96-0.99), and for waist circumference 0.96 (0.95-0.98). A similar trend was shown when the number of teeth was categorised in four categories (0, 1–9, 10–19, ≥20) and used as the independent variable. However, the statistical significance for WHR and for BMI was borderline in the full models (covariates: age, SES, lifestyle, dental anxiety and co-morbidity), but remained statistically significant for waist circumference. When a dichotomised variable (0–19 teeth versus ≥20 teeth) was used, the associations remained statistically significant for all three obesity measures, also when accounting for possible confounders. Furthermore, a larger number of restored teeth showed a statistically significant lower risk of obesity however, borderline significant for BMI and waist circumference but not significant for WHR in the full models. On the contrary, carious teeth revealed no associations with obesity.

**Table 3:** Associations between independent dental variables and obesity

**Independent variable**	**Adjustment**	**BMI**	**WHR**	**Waist**
		**OR (95% CI)**	**OR (95% CI)**	**OR (95% CI)**
No of teeth (continuous variable)	Crude	0.96 (0.95-0.98)	0.97 (0.96-0.99)	0.96 (0.95-0.98)
	Age	0.97 (0,95-0.99)	0.98 (0.96-0.99)	0.97 (0.95-0.99)
	Age, SES, lifestyle, dental anxiety, co-morbidity	0.97 (0.95-1.00)	0.98 (0.96-1.00)	0.97 (0.95-0.99)
No of teeth (categorized: 0, 1–9, 10–19; reference: ≥20)	Crude	1.35 (1.17-1.56)	1.25 (1.09-1.42)	1.38 (1.21-1.56)
	Age	1.29 (1.10-1.52)	1.18 (1.02-1.36)	1.27 (1.11-1.46)
	Age, SES, lifestyle, dental anxiety, co-morbidity	1.22 (1.01-1.48)	1.19 (1.00-1.42)	1.25 (1.06-1.47)
No of teeth (<20 teeth, reference: ≥20 teeth)	Crude	1.95 (1.40-2.73)	1.67 (1.28-2.19)	1.94 (1.47-2.55)
	Age	1.78 (1.23-2.56)	1.51 (1.13-2.02)	1.61 (1.20-2.18)
	Age, SES, lifestyle, dental anxiety, co-morbidity	1.67 (1.10-2.55)	1.48 (1.05-2.07)	1.60 (1.13-2.28)
No of restored teeth* (continuous variable)	Crude	0.96 (0.93-0.98)	0.98 (0.96-1.00)	0.97 (0.95-0.99)
	Age	0.96 (0.94-0.99)	0.99 (0.96-1.01)	0.97 (0.95-0.99)
	Age, SES, lifestyle, dental anxiety, co-morbidity	0.97 (0.94-1.00)	0.99 (0.96-1.01)	0.98 (0.95-1.00)
Teeth with carious lesions* (yes, reference: no)	Crude	1.02 (0.63-1.65)	1.37 (0.92-2.01)	1.25 (0.85-1.84)
	Age	0.98 (0.60-1.59)	1.32 (0.90-1.95)	1.19 (0.80-1.75)
	Age, SES, lifestyle, dental anxiety, co-morbidity	0.78 (0.45-1.36)	1.34 (0.86-2.11)	0.81 (0.51-1.28)
Latest dental visit (≥1 year, reference: <1 year)	Crude	1.76 (1.21-2.56)	1.23 (0.88-1.71)	1.58 (1.14-2.19)
	Age	1.63 (1.12-2.38)	1.13 (0.81-1.58)	1.41 (1.02-1.97)
	Age, SES, lifestyle, dental anxiety, co-morbidity	1.42 (0.91-2.22)	1.18 (0.79-1.76)	1.29 (0.87-1.92)
Dental visit habits (<once a year, reference: ≥once a year)	Crude	1.83 (1.23-2.71)	1.29 (0.90-1.85)	1.96 (1.39-2.75)
	Age	1.64 (1.10-2.46)	1.16 (0.81-1.67)	1.70 (1.20-2.41)
	Age, SES, lifestyle, dental anxiety, co-morbidity	1.28 (0.79-2.09)	1.20 (0.77-1.86)	1.51 (0.99-2.31)
Xerostomia (≥6 months, reference: <6 months)	Crude	1.67 (1.19-2.35)	1.37 (1.02-1.83)	1.60 (1.20-2.14)
	Age	1.55 (1.09-2.19)	1.26 (0.94-1.70)	1.43 (1.06-1.92)
	Age, SES, lifestyle, dental anxiety, co-morbidity	1.33 (0.90-1.98)	1.04 (0.75-1.45)	1.20 (0.86-1.68)
Masticatory function (poor, reference: acceptable)	Crude	1.29 (0.87-1.91)	1.06 (0.76-1.47)	1.11 (0.79-1.55)
	Age	1.23 (0.83-1.83)	1.01 (0.73-1.41)	1.04 (0.74-1.46)
	Age, SES, lifestyle, dental anxiety, co-morbidity	1.16 (0.73-1.83)	0.85 (0.58-1.25)	0.94 (0.63-1.40)

Dependent variables: anthropometric measures BMI ≥30 kg m^2^ , WHR ≥ 0.80 and waist > 88 cm.

Confounders entered in model as specified: SES (socio-economic status = marital status, social and economic situation), lifestyle (tobacco and alcohol use, physical activity, participation in cultural activities), dental anxiety and co-morbidity (diabetes, hypertension, angina pectoris and/or myocardial infarction).

* edentulous individuals excluded (n = 126).

Dental visits, both the time since the most recent visit and regular habits, were associated with BMI and waist circumference, but not with WHR in crude analyses. The associations were modified by the covariates introduced (Table 3). The interaction terms between dental anxiety (DAS) and dental visiting habits (latest and regular) were statistically significant when BMI and waist circumference, respectively, were the dependent variables, but not when using WHR (not in tables). Regarding regular visiting habits, the OR (95% CI) for the term using BMI as the dependent variable was 2.94 (1.21-7.13), for waist circumference 2.48 (1.06-5.78) and for WHR 1.44 (0.56-3.71).

Self-perceived dry mouth, or xerostomia, was associated with obesity in a crude analysis (all three measures) however, the statistical significances disappeared when adjustment was made for possible confounders. Masticatory function showed no associations with obesity, neither crude nor adjusted, and irrespective of the measure of obesity used (Table 3).

Finally, self-perceived general health was introduced as an adjustment factor using different cut-offs for the number of teeth (continuous variable, categorised in four groups, dichotomised 0–19 vs. ≥20 teeth, edentulous vs. dentate) as independent variables and different obesity measures as dependent variables (not in tables). Self-perceived general health then served as a proxy for how general health influences obesity. Irrespective of the number of teeth cut-off level, self-perceived general health was consistently statistically significant when BMI and waist circumference were used as the outcome variable: number of teeth as continuous variable, OR 1.69 (95% CI 1.11-2.57) for BMI and 1.77 (1.26-2.50) for waist circumference, but not for WHR 1.26 (0.94-1.70).

## Discussion

The objective of this study was to describe the associations between oral health variables and total and central adiposity, respectively. The main findings were firstly, that the number of teeth was the factor most consistently associated with obesity in the study population and, secondly, that the choice of obesity measure was of significance for the results. Independent variables representing dental health were more often associated with the obesity indicators of BMI and waist circumference than with WHR.

A strength of the study was the randomised sampling of the female population in a major Swedish city and the fact that all clinical data were collected under standardised conditions [17]. The sample was generated from mainly urban, but also suburban areas. The participation rate was also high, lending support to the representativeness for the current analyses of the subjects of women in Gothenburg in the ages studied [18]. However, generalization to other populations should be made with cautiousness. Also, the participants were middle-aged and elderly, excluding younger adults. The cross-sectional design of the current study precluded conclusions of causal relationships. The directions of the demonstrated associations could therefore not be established.

Tooth loss, i. e. a reduced number of teeth, has previously been found to be related to obesity [4,5]. This was confirmed in the present study where similar analyses generated strong and the most consistent associations. The findings were independent of the categorisation of the number of teeth and also independent of the obesity measure used. A number of common behavioural and biological risk factors for obesity and oral diseases have been observed [3]. Recent research found that one possible factor is inflammation that might be the intermediate factor between obesity and poor dental outcomes, such as periodontitis resulting in tooth loss [23-25]. Moreover, a larger number of restored teeth implied a lower probability of obesity. The mechanism behind this might be a greater overall interest of those subjects taking care of their health. Also, the restorations might have been quite old and the teeth with more severe decay had been removed. However, the opposite that is, having unattended carious lesions, could not be related to any of the obesity measures. The timing of the introduction of fluoride prevention in the later decades might also be influential. Thus, the younger women would be expected to have had more protection from fluoride even if they had a tendency to obesity. Divergent findings regarding the relationship between obesity and dental caries indicate a need for further studies in the field [3,26]. Self-perceived mouth dryness was related to obesity, in accordance with the findings in one other study [27] however, this association was modified by other factors in the full models.

The probability of regular use of dental health services was lower among obese than among non-obese participants in the study, which is in accordance with another Swedish study [5]. Dental service use is related to other health habits, which might explain this association [13]. This was supported by the finding that the interaction between dental anxiety and dental visiting habits was significant, in accordance with one other study [5]. The self-perceived general health, i e an aspect of quality of life, was also lower in this group which is concordant with other studies in obese people [28,29].

Associations between low socio-economic status and being overweight have been demonstrated [14,16]. However, in a report from The Prospective Population Study of Women in Gothenburg, Cabrera et al. [30] found that the associations between tooth loss and cardiovascular disease and cancer, respectively, were stable and independent of socio-economic status. Likewise, the number of missing teeth was related to ischemic heart disease with a minor influence of other variables [31]. This was in accordance with the findings in the present study, where the impact of these possible confounders on the statistical models was minor and almost unchanged when BMI and waist circumference were used as outcome measures. Still, in our study the adjustments were more influential in models using WHR as the dependent variable. This ratio may mirror other aspects of body composition than mere adiposity for instance, a large hip circumference may indicate large muscles [7]. Moreover, the measure of obesity was significant, particularly regarding dental behaviour with a common tendency towards a greater risk with a high BMI and large waist circumference but not with a high WHR. Earlier studies on general morbidity and mortality found that the choice of obesity measure was of similar significance [7,32]. Our findings correspond with these studies, emphasizing a corresponding importance of chosen obesity measure in oral health investigations.

## Conclusions

In conclusion, associations were found in this study between oral health and obesity. General and oral health promotion should occur in parallel, since common risk behaviours can be targeted. The choice of obesity measure should be carefully considered and further investigated in oral health studies.

## Competing interests

The authors report no conflicts of interest. The authors alone are responsible for the content and writing of the paper.

## Authors’ contributions

ALÖ contributed to the design of this study, carried out the statistical analyses and drafted the manuscript. CB was involved in acquisition of data, contributed with the study idea and critical review of the manuscript. LL was involved in acquisition of data and contributed with critical review of the manuscript. MH contributed in the development of the study design, provided biostatistical expertise for data analyses and critical review of the manuscript. All the authors have read and approved the final manuscript.

## Pre-publication history

The pre-publication history for this paper can be accessed here:

http://www.biomedcentral.com/1472-6831/12/50/prepub
